# Supplementary material for: Text classification models for assessing the completeness of randomized controlled trial publications based on CONSORT reporting guidelines
Source: Sci Rep. 2024 Sep 17;14:21721. doi: 10.1038/s41598-024-72130-7 (PMC11408668; doi:10.1038/s41598-024-72130-7)
Supplement: Supplementary file 1 — Supplementary Information. [file 41598_2024_72130_MOESM1_ESM.docx]

**Supplementary Material for** ***Text classification models for assessing the completeness of randomized controlled trial publications based on CONSORT reporting guidelines***

Lan Jiang, MS, Mengfei Lan, MS, Joe D. Menke, MS, Colby J Vorland, PhD, and Halil Kilicoglu, PhD

1. **CONSORT checklist**

The CONSORT checklist items and their descriptions are provided below in Table S1. We refer the reader to Moher et al.^1^ for reporting examples of each item.

**Table S1.** CONSORT checklist items, corresponding item numbers, and the sections to which they typically belong.

| **Section** | **Item** | **Item No** | **Description** |
| --- | --- | --- | --- |
| Title |  | 1a | Identification as a randomized trial in the title |
| Abstract |  | 1b | Structured summary of trial design, methods, results, and conclusions |
| Introduction | Background | 2a | Scientific background and explanation of rationale |
|  | Objectives | 2b | Specific objectives or hypotheses |
| Methods | Trial design | 3a | Description of trial design (such as parallel, factorial) including allocation ratio |
|  |  | 3b | Important changes to methods after trial commencement (such as eligibility criteria), with reasons |
|  | Participants | 4a | Eligibility criteria for participants |
|  |  | 4b | Settings and locations where the data were collected |
|  | Interventions | 5 | Interventions for each group with sufficient details to allow replication, including how and when they were administered |
|  | Outcomes | 6a | Completely defined pre-specified primary and secondary outcome measures, including how and when they were assessed |
|  |  | 6b | Any changes to trial outcomes after the trial commenced, with reasons |
|  | Sample size | 7a | How sample size was determined |
|  |  | 7b | When applicable, explanation of any interim analyses and stopping guidelines |
|  | Randomization: Sequence generation | 8a | Method used to generate the random allocation sequence |
|  |  | 8b | Type of randomization: details of any restriction (such as blocking and block size) |
|  | Randomization: Allocation concealment | 9 | Mechanism used to implement the random allocation sequence (such as sequentially numbered containers), describing any steps taken to conceal the sequence until interventions were assigned |
|  | Randomization: Implementation | 10 | Who generated the random allocation sequence, who enrolled participants, and who assigned participants to interventions |
|  | Blinding | 11a | If done, who was blinded after assignment to interventions (for example, participants, care providers, those assessing outcomes) and how |
|  |  | 11b | If relevant, description of the similarity of interventions |
|  | Statistical methods | 12a | Methods used to compare groups for primary and secondary outcomes |
|  |  | 12b | Methods for additional analyses, such as subgroup analyses and adjusted analyses |
| Results | Participant flow | 13a | For each group, the number of participants who were randomly assigned, received intended treatment, and were analyzed for the primary outcome |
|  |  | 13b | For each group, losses and exclusions after randomization, together with reasons |
|  | Recruitment | 14a | Dates defining the periods of recruitment and follow-up |
|  |  | 14b | Why the trial ended or was stopped |
|  | Baseline data | 15 | A table showing baseline demographic and clinical characteristics for each group |
|  | Numbers analyzed | 16 | For each group, number of participants included in each analysis and whether the analysis was by original assigned groups |
|  | Outcomes and estimation | 17a | For each primary and secondary outcome, results for each group, and the estimated effect size and its precision (such as 95% confidence interval) |
|  |  | 17b | For binary outcomes, presentation of both absolute and relative effect sizes |
|  | Ancillary analyses | 18 | Results of any other analyses performed, including subgroup analyses and adjusted analyses, distinguishing pre-specified from exploratory |
|  | Harms | 19 | All important harms and unintended effects in each group |
| Discussion | Limitations | 20 | Trial limitations, addressing sources of potential bias, imprecision, and if relevant, multiplicity of analyses |
|  | Generalizability | 21 | Generalizability (external validity, applicability) of the trial findings |
|  | Interpretation | 22 | Interpretation consistent with results, balancing benefits and harms, and considering other relevant evidence |
| Other | Registration | 23 | Registration number and name of trial registry |
|  | Protocol | 24 | Where the full trial protocol can be accessed, if available |
|  | Funding | 25 | Sources of funding and other support (such as supply of drugs), role of funders |

1. **Prompt for GPT-4 in-context learning**

The GPT-4 prompt for in-context learning consists of the following ordered elements:

- **Task:** A short paragraph that explains the task, describes the input format, and constrains the response format. The prompt for this part is as follows:

“You are an expert at reviewing scientific articles for transparency. I am providing you a CONSORT checklist table, and a list of lists of the sentence id number, section header, and sentence from the article. Based on that CONSORT table, only return a Python list of lists (no additional text, and no html returns needed) with the sentence id number and which CONSORT Item No you would match it to (as a string). Be strict with your matches. Some sentences may have multiple labels (if so, separate them by a comma), and some may have none (in those cases, make it a [‘0’]).

- **Guidelines:** The CONSORT checklist, including related section, item number, and detailed description of each item.
- **Examples:** We experiment with zero-shot, one-shot and five-shot settings. In zero-shot setting, no examples are provided; in-context learning only relies on task definition and guidelines. In one-shot setting, we pick one sentence randomly from the samples in CONSORT-TM for each label. In five-shot setting, we randomly select five examples.
- **Article:** We add the entire article after the task definition, guideline description, and examples.

1. **Item-level results**

**Table S2:** Sentence-level and article-level performance of the best-performing PubMedBERT model (2b-25) and the rule-based method (1a and 1b). The mean and standard deviation of precision, recall, micro-F_1_, and macro-F1 scores over five folds in cross-validation are reported. The difference between *Article (ANY)* and *Article (1+)* is that the latter requires that the model identifies at least one correct sentence for a checklist item. Standard deviation is shown in parentheses.

| **CONSORT item** | **Sentence-Level** | | | **Article (ANY)** | | | **Article (1+)** | | |
| --- | --- | --- | --- | --- | --- | --- | --- | --- | --- |
|  | **Precis.** | **Recall** | **F_1_** | **Precis.** | **Recall** | **F_1_** | **Precis.** | **Recall** | **F_1_** |
|  | **-** | **-** | **-** |  |  |  |  |  |  |
| Title Randomized (1a) | - | - | - | 1.00 | 1.00 | 1.00 | - | - | - |
| Structured Abstract (1b) | - | - | - | 1.00 | 1.00 | 1.00 | - | - | - |
| Objectives (2b) | 0.90 (0.08) | 0.88 (0.05) | 0.88 (0.05) | 1.00 (0.00) | 0.94 (0.05) | 0.97 (0.03) | 1.00 (0.00) | 0.94 (0.05) | 0.97 (0.03) |
| Trial Design (3a) | 0.82 (0.12) | 0.69 (0.09) | 0.73 (0.05) | 0.98 (0.05) | 0.78 (0.07) | 0.86 (0.05) | 0.98 (0.05) | 0.78 (0.07) | 0.86 (0.05) |
| Changes to Trial Design (3b) | 0.00 (0.00) | 0.00 (0.00) | 0.00 (0.00) | 0.00 (0.00) | 0.00 (0.00) | 0.00 (0.00) | 0.00 (0.00) | 0.00 (0.00) | 0.00 (0.00) |
| Eligibility Criteria (4a) | 0.91 (0.08) | 0.86 (0.05) | 0.88 (0.02) | 1.00 (0.00) | 0.96 (0.05) | 0.98 (0.03) | 1.00 (0.00) | 0.96 (0.05) | 0.98 (0.03) |
| Data Collection Setting (4b) | 0.75 (0.07) | 0.72 (0.12) | 0.72 (0.05) | 0.95 (0.10) | 0.78 (0.11) | 0.84 (0.07) | 0.93 (0.10) | 0.78 (0.11) | 0.83 (0.06) |
| Interventions (5) | 0.75 (0.05) | 0.74 (0.07) | 0.74 (0.04) | 1.00 (0.00) | 0.94 (0.08) | 0.97 (0.04) | 0.96 (0.05) | 0.94 (0.08) | 0.95 (0.04) |
| Outcomes (6a) | 0.83 (0.04) | 0.86 (0.08) | 0.84 (0.06) | 1.00 (0.00) | 1.00 (0.00) | 1.00 (0.00) | 1.00 (0.00) | 1.00 (0.00) | 1.00 (0.00) |
| Changes to Outcomes (6b) | 0.00 (0.00) | 0.00 (0.00) | 0.00 (0.00) | 0.00 (0.00) | 0.00 (0.00) | 0.00 (0.00) | 0.00 (0.00) | 0.00 (0.00) | 0.00 (0.00) |
| Sample Size Determination (7a) | 0.91 (0.07) | 0.88 (0.08) | 0.89 (0.06) | 1.00 (0.00) | 1.00 (0.00) | 1.00 (0.00) | 1.00 (0.00) | 1.00 (0.00) | 1.00 (0.00) |
| Interim Analyses/ Stopping Guidelines (7b) | 0.88 (0.13) | 0.38 (0.05) | 0.52 (0.02) | 0.83 (0.17) | 0.58 (0.08) | 0.67 (0.00) | 0.83 (0.17) | 0.58 (0.08) | 0.67 (0.00) |
| Sequence Generation (8a) | 0.81 (0.13) | 0.72 (0.18) | 0.76 (0.16) | 0.94 (0.08) | 0.82 (0.14) | 0.87 (0.10) | 0.87 (0.15) | 0.80 (0.17) | 0.83 (0.14) |
| Randomization Type (8b) | 0.79 (0.12) | 0.74 (0.12) | 0.75 (0.09) | 0.97 (0.06) | 0.84 (0.05) | 0.90 (0.05) | 0.97 (0.06) | 0.84 (0.05) | 0.90 (0.05) |
| Allocation Concealment (9) | 0.59 (0.35) | 0.35 (0.13) | 0.40 (0.17) | 0.73 (0.23) | 0.53 (0.25) | 0.56 (0.12) | 0.62 (0.32) | 0.50 (0.26) | 0.46 (0.13) |
| Randomization Implementation (10) | 0.69 (0.20) | 0.59 (0.15) | 0.62 (0.15) | 0.84 (0.11) | 0.84 (0.14) | 0.83 (0.09) | 0.78 (0.15) | 0.84 (0.14) | 0.79 (0.09) |
| Blinding (11a) | 0.77 (0.12) | 0.64 (0.15) | 0.70 (0.13) | 1.00 (0.00) | 0.78 (0.06) | 0.88 0.04) | 0.93 (0.09) | 0.77 (0.06) | 0.84 (0.06) |
| Similarity of Interventions (11b) | 0.55 (0.25) | 0.49 (0.29) | 0.51 (0.27) | 0.88 (0.15) | 0.60 (0.21) | 0.70 (0.16) | 0.82 (0.26) | 0.57 (0.24) | 0.66 (0.23) |
| Statistical Methods for Outcomes (12a) | 0.73 (0.05) | 0.84 (0.04) | 0.78 (0.04) | 1.00 (0.00) | 1.00 (0.00) | 1.00 (0.00) | 1.00 (0.00) | 1.00 (0.00) | 1.00 (0.00) |
| Statistical Methods for Other Analyses (12b) | 0.36 (0.15) | 0.28 (0.12) | 0.30 (0.11) | 0.70 (0.10) | 0.68 (0.21) | 0.67 (0.12) | 0.51 (0.18) | 0.62 (0.21) | 0.51 (0.10) |
| Participant Flow (13a) | 0.75 (0.08) | 0.79 (0.08) | 0.76 (0.01) | 0.98 (0.04) | 0.98 (0.04) | 0.98 (0.04) | 0.96 (0.05) | 0.98 (0.04) | 0.96 (0.03) |
| Participant Loss/Exclusion (13b) | 0.74 (0.07) | 0.69 (0.14) | 0.70 (0.09) | 0.98 (0.04) | 0.93 (0.05) | 0.95 (0.04) | 0.88 (0.08) | 0.92 (0.06) | 0.90 (0.07) |
| Periods of Recruitment/Follow-Up (14a) | 0.88 (0.11) | 0.79 (0.14) | 0.82 (0.04) | 1.00 (0.00) | 0.90 (0.05) | 0.95 (0.03) | 0.98 (0.05) | 0.90 (0.05) | 0.94 (0.04) |
| Trial Stopping (14b) | 0.65 (0.30) | 0.75 (0.32) | 0.59 (0.23) | 0.77 (0.29) | 0.90 (0.20) | 0.80 (0.24) | 0.77 (0.29) | 0.90 (0.20) | 0.80 (0.24) |
| Baseline Data (15) | 0.82 (0.06) | 0.82 (0.12) | 0.81 (0.07) | 1.00 (0.00) | 0.98 (0.04) | 0.99 (0.02) | 1.00 (0.00) | 0.98 (0.04) | 0.99 (0.02) |
| Numbers Analyzed (16) | 0.55 (0.21) | 0.43 (0.17) | 0.47 (0.18) | 0.98 (0.05) | 0.87 (0.10) | 0.92 (0.06) | 0.70 (0.20) | 0.81 (0.14) | 0.74 (0.18) |
| Outcome Results (17a) | 0.68 (0.10) | 0.73 (0.03) | 0.70 (0.05) | 1.00 (0.00 | 0.96 (0.05) | 0.98 (0.03) | 1.00 (0.00 | 0.96 (0.05) | 0.98 (0.03) |
| Binary Outcome Results (17b) | 0.42 (0.14) | 0.41 (0.16) | 0.39 (0.13) | 0.83 (0.10) | 0.98 (0.04) | 0.89 (0.04) | 0.70 (0.11) | 0.97 (0.06) | 0.81 (0.07) |
| Ancillary Analyses (18) | 0.51 (0.14) | 0.40 (0.08) | 0.43 (0.06) | 0.84 (0.12) | 0.90 (0.08) | 0.86 (0.06) | 0.74 (0.14) | 0.89 (0.09) | 0.80 (0.05) |
| Harms (19) | 0.64 (0.05) | 0.72 (0.03) | 0.68 (0.03) | 1.00 (0.00) | 0.87 (0.08) | 0.93 (0.04) | 0.97 (0.06) | 0.86 (0.09) | 0.91 (0.07) |
| Limitations (20) | 0.70 (0.06) | 0.73 (0.02) | 0.72 (0.03) | 0.98 (0.05) | 0.98 (0.05) | 0.98 (0.05) | 0.98 (0.05) | 0.98 (0.05) | 0.98 (0.05) |
| Generalizability (21) | 0.58 (0.18) | 0.37 (0.08) | 0.44 (0.09) | 0.93 (0.13) | 0.54 (0.05) | 0.68 (0.07) | 0.73 (0.23) | 0.47 (0.10) | 0.57 (0.14) |
| Interpretation (22) | 0.68 (0.04) | 0.69 (0.02) | 0.68 (0.02) | 1.00 (0.00) | 1.00 (0.00) | 1.00 (0.00) | 1.00 (0.00) | 1.00 (0.00) | 1.00 (0.00) |
| Registration (23) | 0.91 (0.08) | 0.87 (0.10) | 0.88 (0.07) | 1.00 (0.00) | 0.93 (0.06) | 0.96 (0.03) | 1.00 (0.00) | 0.93 (0.06) | 0.96 (0.03) |
| Protocol Access (24) | 1.00 (0.00) | 0.77 (0.29) | 0.83 (0.21) | 1.00 (0.00) | 0.80 (0.24) | 0.87 (0.16) | 1.00 (0.00) | 0.80 (0.24) | 0.87 (0.16) |
| Funding (25) | 0.77 (0.08) | 0.83 (0.14) | 0.79 (0.07) | 1.00 (0.00) | 1.00 (0.00) | 1.00 (0.00) | 1.00 (0.00) | 1.00 (0.00) | 1.00 (0.00) |
| MICRO | 0.72 (0.02) | 0.71 (0.02) | 0.71 (0.02) | 0.96 (0.02) | 0.89 (0.02) | 0.92 (0.01) | 0.92 (0.02) | 0.88 (0.02) | 0.90 (0.01) |
| MACRO | 0.71 (0.04) | 0.66 (0.03) | 0.67(0.02) | 0.92 (0.04) | 0.85 (0.02) | 0.87 (0.03) | 0.87 (0.04) | 0.84 (0.02) | 0.84 (0.03) |

**Table S3:** Item-level results for the fine-tuned BioGPT model.

| **CONSORT Item** | **Precision** | **Recall** | **F1** |
| --- | --- | --- | --- |
| Objectives (2b) | 0.85 | 0.85 | 0.85 |
| Trial Design (3a) | 0.80 | 0.50 | 0.62 |
| Changes to Trial Design (3b) | 0.00 | 0.00 | 0.00 |
| Eligibility Criteria (4a) | 0.83 | 0.97 | 0.90 |
| Data Collection Setting (4b) | 1.00 | 0.91 | 0.95 |
| Interventions (5) | 0.88 | 0.75 | 0.81 |
| Outcomes (6a) | 0.78 | 0.96 | 0.86 |
| Changes to Outcomes (6b) | 0.00 | 0.00 | 0.00 |
| Sample Size Determination (7a) | 0.88 | 0.92 | 0.90 |
| Interim Analyses/ Stopping Guidelines (7b) | 0.00 | 0.00 | 0.00 |
| Sequence Generation (8a) | 0.60 | 0.67 | 0.63 |
| Randomization Type (8b) | 0.75 | 1.00 | 0.86 |
| Allocation Concealment (9) | 1.00 | 0.25 | 0.40 |
| Randomization Implementation (10) | 0.54 | 0.70 | 0.61 |
| Blinding (11a) | 0.67 | 0.25 | 0.36 |
| Similarity of Interventions (11b) | 0.00 | 0.00 | 0.00 |
| Statistical Methods for Outcomes (12a) | 0.61 | 0.81 | 0.70 |
| Statistical Methods for Other Analyses (12b) | 0.30 | 0.13 | 0.18 |
| Participant Flow (13a) | 0.69 | 0.67 | 0.68 |
| Participant Loss/Exclusion (13b) | 0.71 | 0.55 | 0.62 |
| Periods of Recruitment/Follow-Up (14a) | 0.70 | 0.64 | 0.67 |
| Trial Stopping (14b) | 0.00 | 0.00 | 0.00 |
| Baseline Data (15) | 0.83 | 0.78 | 0.80 |
| Numbers Analyzed (16) | 0.56 | 0.20 | 0.29 |
| Outcome Results (17a) | 0.72 | 0.69 | 0.70 |
| Binary Outcome Results (17b) | 0.30 | 0.52 | 0.38 |
| Ancillary Analyses (18) | 0.31 | 0.22 | 0.26 |
| Harms (19) | 0.56 | 0.74 | 0.64 |
| Limitations (20) | 0.67 | 0.65 | 0.66 |
| Generalizability (21) | 0.75 | 0.38 | 0.50 |
| Interpretation (22) | 0.63 | 0.70 | 0.66 |
| Registration (23) | 0.91 | 0.91 | 0.91 |
| Protocol Access (24) | 0.00 | 0.00 | 0.00 |
| Funding (25) | 0.79 | 0.71 | 0.75 |
| MICRO | 0.68 | 0.68 | 0.68 |
| MACRO | 0.62 | 0.57 | 0.58 |

**Table S4:** Item-level results of zero-shot GPT-4 direct inference. Note that these results are obtained from 10 articles.

| **CONSORT Item** | **Precision** | **Recall** | **F1** |
| --- | --- | --- | --- |
| Objectives (2b) | 0.19 | 0.71 | 0.30 |
| Trial Design (3a) | 0.43 | 0.82 | 0.56 |
| Changes to Trial Design (3b) | 0.00 | 0.00 | 0.00 |
| Eligibility Criteria (4a) | 0.73 | 0.97 | 0.83 |
| Data Collection Setting (4b) | 0.46 | 0.55 | 0.50 |
| Interventions (5) | 0.66 | 0.70 | 0.68 |
| Outcomes (6a) | 0.70 | 0.50 | 0.58 |
| Changes to Outcomes (6b) | 0.00 | 0.00 | 0.00 |
| Sample Size Determination (7a) | 0.73 | 1.00 | 0.85 |
| Interim Analyses/ Stopping Guidelines (7b) | 1.00 | 1.00 | 1.00 |
| Sequence Generation (8a) | 0.64 | 0.90 | 0.75 |
| Randomization Type (8b) | 0.64 | 0.64 | 0.64 |
| Allocation Concealment (9) | 0.64 | 0.88 | 0.74 |
| Randomization Implementation (10) | 0.50 | 0.27 | 0.35 |
| Blinding (11a) | 0.64 | 0.70 | 0.67 |
| Similarity of Interventions (11b) | 0.50 | 0.50 | 0.50 |
| Statistical Methods for Outcomes (12a) | 0.67 | 0.56 | 0.61 |
| Statistical Methods for Other Analyses (12b) | 0.22 | 0.30 | 0.26 |
| Participant Flow (13a) | 0.53 | 0.83 | 0.65 |
| Participant Loss/Exclusion (13b) | 0.38 | 0.17 | 0.23 |
| Periods of Recruitment/Follow-Up (14a) | 0.60 | 0.5 | 0.55 |
| Trial Stopping (14b) | 0.00 | 0.00 | 0.00 |
| Baseline Data (15) | 0.90 | 0.77 | 0.83 |
| Numbers Analyzed (16) | 0.35 | 0.31 | 0.33 |
| Outcome Results (17a) | 0.40 | 0.41 | 0.41 |
| Binary Outcome Results (17b) | 0.00 | 0.00 | 0.00 |
| Ancillary Analyses (18) | 0.32 | 0.32 | 0.32 |
| Harms (19) | 0.25 | 0.95 | 0.40 |
| Limitations (20) | 0.36 | 0.48 | 0.41 |
| Generalizability (21) | 0.29 | 0.71 | 0.42 |
| Interpretation (22) | 0.52 | 0.42 | 0.47 |
| Registration (23) | 1.00 | 0.83 | 0.91 |
| Protocol Access (24) | 0.07 | 1.00 | 0.13 |
| Funding (25) | 0.71 | 0.96 | 0.82 |
| MICRO | 0.48 | 0.54 | 0.51 |
| MACRO | 0.47 | 0.58 | 0.49 |

1. **Data augmentation analysis**

Some data augmentation outcomes are provided in Table S5. GPT-4 generative method mainly uses checklist item description, so the generated sentence has little overlap with the original sentence in this case. GPT-4 rephrasing largely preserves the original meaning. EDA and UMLS-EDA insert tokens that lead to incoherent meaning. Overall, GPT-4 rephrasing, EDA, and UMLS-EDA introduce little diversity from the training set. While GPT-4 generative enhances diversity compared to existing training examples, generated sentences themselves are less diverse.

**Table S5:** Data augmentation outcomes using different methods. The original sentence used for GPT-4 paraphrasing, EDA, and UMLS-EDA is *The packaging and labeling of the study drug kits were based on a separate drug packaging randomization schedule*, with the label Allocation Concealment Mechanism (9). The item description used for generative augmentation is “*the mechanism used to implement the random allocation sequence, describing any steps taken to conceal the sequence until interventions were assigned, with specifics*”. EDA augmentation randomly inserts a token “*randomization*” into the sentence. UMLS-EDA introduces “*[mental process]*” into the sample.

| **Augmentation method** | **Augmentation result** |
| --- | --- |
| GPT-4 generative | To achieve random allocation, we used a shuffled deck of cards each representing a treatment category, keeping every card faced down until the time came to assign treatment legitimately. |
| GPT-4 rephrasing | A distinct randomization schedule for drug packaging was utilized for the arrangement and tagging of the study drug kits. |
| EDA | The packaging and labeling of the study drug randomization kits were based on a separate drug packaging randomization schedule. |
| UMLS-EDA | The packaging and labeling [mental process] of the study medication kits were based on a separate drugs packaged randomization schedule. |

1. **Model Interpretation**

To gain insights into the model behavior, we applied integrated gradients algorithm^3^ and created saliency maps. We show the saliency maps for three examples accurately classified with the best-performing PubMedBERT model in Table S6. In the first example, keywords *linearity* and *exploratory* receive positive attention from the model for the prediction Statistical Methods for Outcomes (12a), *received* is positively attended to for the prediction Intervention (5) in the second example and *impossible* and *might have* for the prediction Limitations (20) in the third example. In the second and third examples, section headers (*study design and procedures* and *discussion*) also play a positive role. Examples also seem to suggest that the most positively weighted tokens are included in the classified sentence rather than the previous or next sentence.

**Table S6:** Saliency maps for three examples classified accurately by the best-performing PubMedBERT model. Tokens receiving positive attention from the model are marked in green and those receiving negative attention in red. The intensity of color corresponds to the weight of the feature. Section headers in the saliency maps are marked with rectangles around them.

| CONSORT item | Saliency Map |
| --- | --- |
| Statistical Methods for Outcomes (12a) | 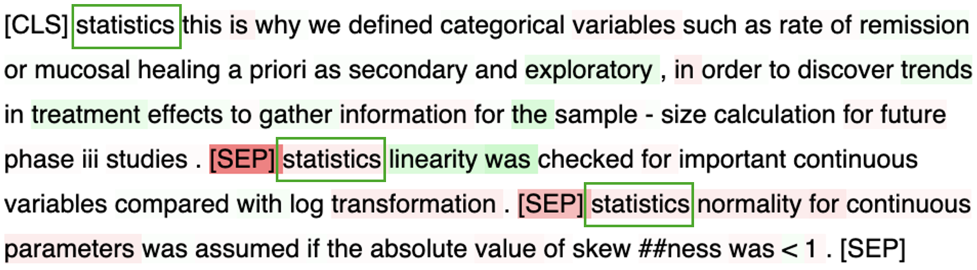 |
| Interventions (5) | 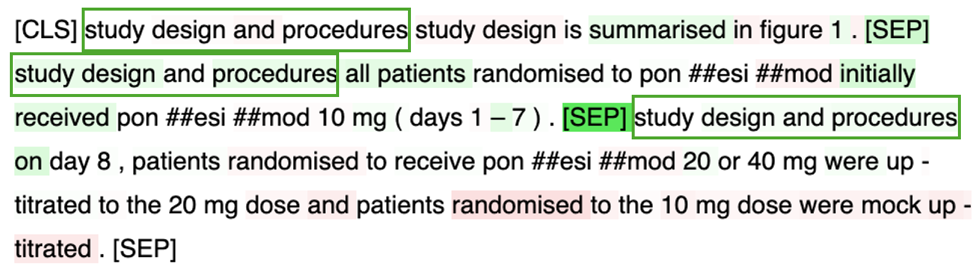 |
| Limitations (20) | 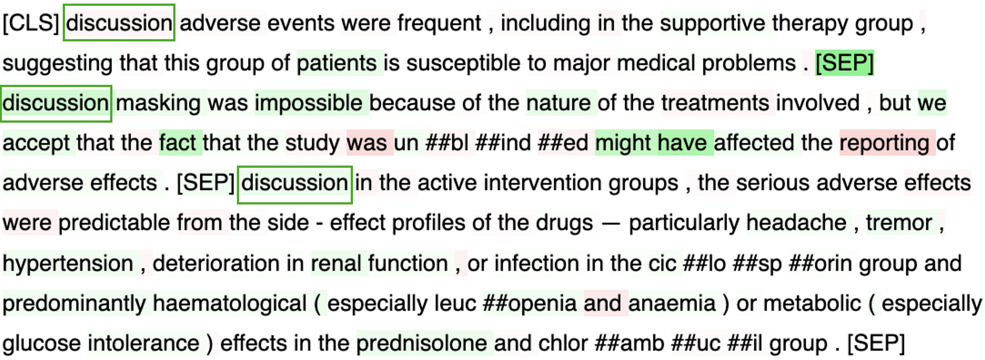 |

**REFERENCES**

1. Moher D, Hopewell S, Schulz KF, Montori V, Gøtzsche PC, Devereaux PJ, et al. CONSORT 2010 Explanation and Elaboration: updated guidelines for reporting parallel group randomised trials. *BMJ*. 2010;340.
2. Kilicoglu H, Rosemblat G, Hoang L, Wadhwa S, Peng Z, Malički M, Schneider J, ter Riet, G. Toward assessing clinical trial publications for reporting transparency. *Journal of Biomedical Informatics*. 2021;116:103717.
3. Sundararajan M, Taly A, Yan Q. Axiomatic attribution for deep networks. In *International conference on machine learning* 2017 (pp. 3319-3328).
